# Piloting a mental health training programme for community health workers in South Africa: an exploration of changes in knowledge, confidence and attitudes

**DOI:** 10.1186/s12888-018-1772-1

**Published:** 2018-06-14

**Authors:** Goodman Sibeko, Peter D. Milligan, Marinda Roelofse, Lezel Molefe, Deborah Jonker, Jonathan Ipser, Crick Lund, Dan J. Stein

**Affiliations:** 1 Department of Psychiatry and Mental Health, University of Cape Town, Cape Town, South Africa; 2 Western Cape Department of Health, Western Cape Province, South Africa; 3 Centre for Global Mental Health, Institute of Psychiatry, Psychology and Neuroscience, King’s College London, London, UK; 4 South African Medical Research Council Unit on Risk and Resilience in Mental Disorders, Cape Town, South Africa

**Keywords:** Task shifting, Community health workers, Mental health, Training

## Abstract

**Background:**

There is a shortage of trained mental health workers in spite of the significant contribution of psychiatric disorders to the global disease burden. Task shifting, through the delegation of health care tasks to less specialised health workers such as community health workers (CHWs), is a promising approach to address the human resource shortage. CHWs in the Western Cape province of South Africa provide comprehensive chronic support which includes that for mental illness, but have thus far not received standardized mental health training. It is unknown whether a structured mental health training programme would be acceptable and feasible, and result improved knowledge, confidence and attitudes amongst CHWs.

**Methods:**

We developed and piloted a mental health training programme for CHWs, in line with the UNESCO guidelines; the WHO Mental Health Gap Action Programme and the South African National framework for CHW training. In our quasi-experimental (before-after) cohort intervention study we measured outcomes at the start and end of training included: 1) Mental health knowledge, measured through the use of case vignettes and the Mental Health Knowledge Schedule; 2) confidence, measured with the Mental Health Nurse Clinical Confidence Scale; and 3) attitudes, measured with the Community Attitudes towards the Mentally Ill Scale. Knowledge measures were repeated 3 months later. Acceptability data were obtained from daily evaluation questionnaires and a training evaluation questionnaire, while feasibility was measured by participant attendance at training sessions.

**Results:**

Fifty-eight CHWs received the training, with most (*n* = 56, 97.0%) attending at least 7 of the 8 sessions. Most participants (*n* = 29, 63.04%) demonstrated significant improvement in knowledge, which was sustained at 3-months. There was significant improvement in confidence, along with changes in attitude, indicating improved benevolence, reduced social restrictiveness, and increased tolerance to rehabilitation of the mentally ill in the community but there was no change in authoritarian attitudes. The training was acceptable and feasible.

**Conclusions:**

Mental health training was successful in improving knowledge, confidence and attitudes amongst trained CHWs. The training was acceptable and feasible. Further controlled studies are required to evaluate the impact of such training on patient health outcomes.

**Trial registration:**

PACTR PACTR201610001834198, Registered 26 October 2016.

## Background

Mental, neurological and substance use disorders contribute significantly to the global burden of disease, accounting for 21.2% of years lived with disability [1]. In spite of this, mental health services in general are comparatively under-resourced, with a total mental health workforce of only 9 per 100,000 population globally, of which 0.9 per 100,000 are psychiatrists [2]. There is an inequitable distribution of human and other resources in low- and middle-income countries, with a total mental health work force of 0,9 per 100,000 populations in low-income countries, 3,2 in lower-middle income countries and 15,9 in upper middle-income countries compared to 52,3 in high-income countries. As a consequence, in low-resource settings, the treatment gap, an indication of the proportion of individuals with mental illness who remain untreated in spite of the existence of effective treatments, is as high as 90% [3, 4].

Task shifting is one approach that seeks to address this treatment gap, and involves the delegation of health care delivery tasks, where appropriate, to less specialized health workers [5]. Interventions employing task shifting may help to make more efficient use of human resources and have yielded some promising results in improving outcomes for mental health service users [6]. Of the four types of task shifting described [5], type 3 involves the delegation of nurses’ and midwives’ tasks to nursing assistants or nursing aides or community health workers (CHWs).

CHWs are defined as “community based workers that help individuals and groups in their own communities to access health and social services, and educate community members about various health issues” [7]. CHWs have shorter training than professional health care workers and need not be part of the organization of a health system but should be supported by it. CHWs in Cape Town, South Africa provide home based preventive_,_ supportive and rehabilitative care under the supervision of various non-profit organizations [8]. Their role includes general medical support with a focus on reducing risk factors that contribute significantly to the burden of disease, which includes support for mental health service users. CHW’s in the Western Cape of South Africa are required to be able to read and write, with no additional education level qualification. These CHWs receive basic training in the provision of chronic care, including that for hypertension, diabetes, tuberculosis and HIV. There is however currently no standard mental health-training or existing training programme for CHWs in this region.

While CHW’s in the Western Cape have responded well to training directed at improving their capacity to deliver general chronic care, it is unclear whether structured CHW training focussed on mental health results in improved knowledge, confidence and a change in attitudes towards mental illness. It is further unclear to what extent such improvement would translate to improved care and support for mental health service users. We therefore developed a locally appropriate structured CHW mental health training programme_,_ in close collaboration with the Western Cape Department of Health, and evaluated the programme’s success in improving the knowledge_,_ skill and confidence amongst trained CHWs. We further evaluated the acceptability and feasibility of the programme_,_ both of which factors have been linked to the success of programmes aimed at CHWs [9]. Due to resource limitations, service user outcomes were not measured in this study. The study was registered with the Pan African Clinical Trials Registry (PACTR201610001834198).

## Methods

Our study design was a quasi-experimental (before-after) cohort intervention study design, which is ideal for the comparing naturalistic units such as CHW groups [10]. Data collected at baseline included participant demographic data; and pre-training data for knowledge, confidence and attitudes_,_ Daily training evaluation data were collected at the end of each training session, detailing feedback about the session. Post-training data for knowledge, confidence and attitudes were collected at the end of the training programme. Knowledge data were collected again 3 months after the conclusion of training. Additionally, on the last day of training, training evaluation data were collected, obtaining feedback about the conduct of the training programme. All data were collected anonymously through the use of a unique identifier_,_ on questionnaires to allow for matching of pre- and post-training data. Any document containing the information linking trainees to their unique identifier was kept confidentially in a password protected spreadsheet.

### Study sites and participants

Recruited participants consisted of CHWs supervised by four Non-Governmental Organizations (NGOs) within the Western Cape, where mental health services are organized as follows: There are four psychiatric hospitals: Alexandra Hospital in Maitland, Lentegeur in Mitchell’s Plain_,_ Stikland Hospital located in Bellville, and Valkenberg Hospital located in Observatory [11]. New Beginnings is a sub-acute facility, which falls under Stikland Hospital’s supervision. William Slater provides a mental health step-down facility under Valkenberg Hospital. These hospitals provide tertiary in- and out-patient psychiatric services_,_ within a network of health facilities that include Regional and District Hospitals_,_ Community Health Centres_,_ and Clinics, ensuring they are embedded within the general care settings and integrated into primary care in line with the South African Mental Health Care Act 17 of 2002 [12]. This allows for mental health care to be delivered at less specialized facilities where possible, while reserving specialized care for those mental health service users who need it. The Western Cape is divided into health districts, which plan, execute and oversee delivery of this integrated care. The health districts are further divided into substructures which, which in addition to being responsible for primary health care_,_ oversee a range of community-based health services. CHWs form an important part of these community-based services and are arranged in groups ranging in size from 20 to 40, supervised by privately-funded NGOs. While the salaries of these CHW’s originates from NGO’s, they are employed under the Western Cape Government’s terms of employment, affording them some employment protection and access to employee wellness programmes. The four selected participating NGO’s are located within four health district sub-structures, which were identified within the Cape Town region for the piloting of the training manual. These were purposively selected by the Western Cape Department of Health. All CHWs in the identified NGOs were eligible to participate.

### Training programme

#### Development

The training programme was guided by the principles outlined in the “UNESCO Training Guide and Training Techniques” and the “Best Practice Guidelines for Implementing and Evaluating CHW Programs in Health Care Settings” documents [13, 14]. We developed the programme in line with the South African National framework for CHWs as set out by the Health and Welfare Sector Education and Training Authority [15].

Development of the training programme began with a review of the processes and experiences of mental health training that had been conducted with a group of CHWs in the Kalkfontein sub-district. This training had been developed by New Beginnings to train CHWs within their sub-district. Details pertaining to the content of this training were provided by the Western Cape Department of Health (WCDoH), and contained feedback from the CHWs who had received this training and from the trainer who had delivered it._,_ The themes covered in the New Beginnings training were then further developed, while drawing on material from the training manual developed by Gibson et al. [16, 17].

The education and intervention guidelines were drawn from the World Health Organization Mental Health Gap Action Programme (WHO mhGAP) Intervention guide [18]. Information pertaining to admission pathways and role players is based on the South African Mental Health Care Act No. 17 of 2002 [12]. The programme takes into consideration locally accepted theories of disease causation and prevailing beliefs as these may impact not only on stigma and health seeking behaviour_,_ but also on clinician symptom appraisal and intervention decision-making [19]. A draft of the training programme was presented to the clinical teams of the Khayelitsha and Mitchell’s Plain health sub-districts for their inputs between February and April 2016, which included specific concerns around language and target proficiency for the CHWs. Adjustments were made accordingly and updated documents shared with all stakeholders at each point.

The first draft of the training program was used to train two groups of CHW’s in June 2016: One group supervised by The Caring Network Khayelitsha (*n* = 20), a NGO in the Khayelitsha substructure and one group supervised by Arisen Women Foundation (*n* = 22), a NGO in the Klipfontein sub-structure. The purpose of this was to evaluate the appropriateness of the content and language and to make final adjustments based on the feedback of the CHWs receiving this initial training.

#### The final programme

The final training programme, consisting of eight 3-h sessions was prepared and piloted in February 2017 [20]. The outline of the programme is presented in Table 1 below. Each session is divided into activities covering specific components. Detailed instructions are provide to assist the facilitator in conducting each activity to ensure that the training objectives of the session are met. The focus of facilitation is on encouraging trainee participation though reflection and discussion. There is significant provision for revision of previously covered content as the programme continues. This final training programme was piloted by training 27 CHW’s supervised by Masincedane in Strand and 31 CHWs supervised by Opportunity To Serve Ministries (OTSM) in Mitchell’s Plain. The CHW supervisors, to whom the CHWs report directly, were part of the training cohort.

**Table 1:** Outline of training

Session	Topic	Elements
1	Introduction and Culture	Ice breaker session, pre-training evaluation forms, and discussion of culture.
2	Culture and Mental Illness	Introduction of mental illness and it’s overlap with local cultural constructs.
3	Mood and Anxiety Disorder	Discussion of the features of these components.
4	Psychotic Disorders, Older People, Intellectual Disabilities, Suicide and Aggression	Discussion of the features of these components and an approach to suicide and aggression.
5	Substance Use Disorders and Management of Mental Illness	Discussion of substance use, abuse and dependence and the management of previously introduced mental illnesses.
6	The Role of the Community Health Worker	Discussion of the role of the Community Health Worker, a review of mental disordered previously discussed, and a discussion of adherence and general support skills
7	The Mental Health Care Act and Admission Pathway	Discussion of the mental health act, evaluation and admission pathways and processes.
8	CHW Experiences, Case Vignettes, Evaluation Forms and Closure	The CHWs reflect on their training and experience in the field, and complete the post training evaluation documents.

### Delivery of the training

The training programme was delivered by a trilingual (English_,_ Xhosa and Afrikaans) social worker (LM) with extensive experience in training and in multidisciplinary mental health service delivery. The training was hosted on-site at the premises of the selected NGO’s. The training was presented with the aid of a PowerPoint presentation. Reflection and discussion was encouraged during the conduct of the training.

### Evaluation and measures

#### Knowledge

We measured mental health knowledge through the use of case vignettes and by using the Mental Health Knowledge Schedule (MAKS) [21]. Case vignettes questions sought to assess diagnostic areas covered in the training, and were designed to elicit the CHWs’ impression of likely diagnosis, appropriate intervention and the role of the CHW in management. The MAKS, which is a widely used measure of mental health knowledge, consists of 12 statements scored on a Likert where a score of 1 corresponds to a scenario where the respondent strongly disagrees with a correct statement and a score of 5 corresponds to a scenario where a respondent strongly agrees with a correct statement [21, 22]. The last 6 statements are intended to measure levels of recognition of various mental health conditions. Item scores were added up to arrive at a total score.

#### Confidence

Confidence in executing mental health care support was measured using the Mental Health Nursing Clinical Confidence Scale (MHNCCS) [23]. The MHNCCS is a self-completed instrument composed of 20 items scored on a Likert scale, where a score of 1 indicates “not at all confident” and a score of 4 indicates complete confidence.

#### Attitudes

Changes in personal attitude towards mental illness were measured using the widely used Community Attitudes Towards The Mentally Ill Scale (CAMI) [22, 24]. There are 4 clusters for the CAMI: authoritarianism, benevolence, social restrictiveness and Community Mental Health Ideology, which indicates tolerance to rehabilitation in the community, divided into an equal number of positively and negatively worded items.

#### Acceptability and feasibility

Acceptability was evaluated through the use of daily evaluation questionnaires and a training evaluation questionnaire. The daily evaluation questionnaires consisted of the following five questions: 1) “What did you enjoy most about today?”; 2) “What did you learn today that you feel will help you in your work?”; 3) “Was there anything you did not understand today’? Please give some examples?”; 4) “What did you learn today that was most important to you?”; 5) “Do you have any other comments about today’s session?”. The qualitative semi-structured Training Evaluation Questionnaire enquired about the content of the training_,_ the tools and processes used during training_,_ the performance of the trainer_,_ the training setting_,_ the usefulness of the training_,_ as well as an overall impression of the training. Feasibility was established through the participant attendance at training sessions.

#### Evaluation procedures

The knowledge, confidence and attitudes questionnaires were conducted at the beginning of training and again at the end of the training. Daily evaluation questionnaires were conducted at the end of every training session. Case vignettes and the Mental Health Knowledge Questionnaire were repeated three months after the end of the training to determine knowledge retention. At both assessment points, case vignettes were conducted first and then collected before the rest of the questionnaires were handed out and completed in order to ensure that case vignette responses were not modified by the content in the questionnaires. Daily evaluation forms were completed by the CHWs at the end of each training. Participating CHWs completed the training evaluation on the last day of training.

### Analysis

The primary outcomes were 1) change in the CHW’s mental health knowledge; 2) change in CHW confidence in supporting mental health service users and 3) change in attitudes towards mental illness, amongst the trained CHWs. We used t tests and regression models to test changes in mean questionnaire scores before and after completion of the training_,_ adjusting for baseline scores. Predictors of the outcomes of interest (changes from pre to post intervention on mean scores for the MAKS, MHNCCS and CAMI) were identified by means of a logistic regression modelling approach, in which the outcomes were regressed individually on a set of basic variables hypothesised to be influential (pre-intervention knowledge_,_ age_,_ education and years of service). Pre-intervention knowledge was included as the first predictor variable in all logistic and linear regression models. A series of exploratory models were subsequently conducted in which variables were added individually to a term representing pre-intervention knowledge, in addition to any significant predictors that emerged from the basic model. These variables included site, being in a stable partnership_,_ having children, having dependents and having a medical condition.

For the case vignettes, differences pre-post the intervention were dichotomized, denoting whether a CHW improved (coded as 1) or not (coded as 0). Differences pre-post on all the other outcomes of interest (including the MAKS, MHNCCS and CAMI) were tested using linear multiple regression models. The negative items of the CAMI were reverse-scored by subtracting each item’s score from the maximum score possible + 1, as this is standard practice. In the case of the CAMI, the max score + 1 = 6, so if a negative item is scored 2, this procedure will result in a score of 4 (6–2). The negatively and positively worded items were then added up for each cluster to get a total score for each.

We provide odds ratios and 95% confidence intervals for logistic regression models in which the outcome is binary (e.g. for the case vignette diagnosis), where the odds ratio would be interpreted as the odds that a person improved at the end of the training. In reporting the results of the multiple linear regression models we have provided coefficients, t and *p* values. Data were entered and analysed using the R statistical computing platform (version 3.4.1) [25].

Qualitative data collected at the end of each training session was managed and analysed using NVIVO 8, following which an inductive thematic analysis approach was adopted [26]. Data were collected at the end of every training session using daily evaluation questionnaires, and at the end of the training programme via a freeform entry field within the training evaluation questionnaire. Two investigators (GS and DJ) familiarized themselves with the data and generated codes. Once codes were reviewed and agreed upon, they were grouped into potential themes, which were then defined and named, and which are presented below.

## Results

### Participants

The characteristics of the participants are presented in Table 2 below. Fifty-eight CHWs participated in the study (31 from Masincedane and 27 from OTSM). None of the participants reported having their own psychiatric illness or having someone in their home with a psychiatric illness. Their CHW work was in general their only employment and source of income. Forty (69.0%) of the 58 CHW’s enrolled into the training attended all 8 sessions, while 15 (26.0%) attended 7 of the 8 sessions. Only 1 (1.7%) attended 6 sessions, while two CHW’s dropped out during the course of the training due to overlapping training commitments, attending 1 and 5 sessions respectively.

**Table 2:** Participant characteristics

Characteristic	Masincedane (*N* = 31) (Mean, SD)	OTSM (*N* = 27) (Mean, SD)
Age in years	32.3 (7.72)	41.48 (12.57)
Years of service as CHW	3.86 (3.94)	2.79 (2.44)
Highest Level of education in grades	11 (0.96)	10.81 (1.4)
Children	1.96 (1.16)	1.9 (1.16)
Dependents^a^	4.56 (3.71)	3.06 (2.34)
	**%**	**%**
Stable partnership^b^	40.74%	58.06%
Has own medical condition	22.22%	41.94%

^a^Dependents refers to total of dependents of different age categories (< 5_,_ 5–18_,_18+)

^b^Stable partnership refers to any marital_,_ customary or committed relationship

### Training outcomes

#### Knowledge

Of 46 individuals with complete pre-and post-intervention data, a total of 29 (63.04%) improved in accuracy of diagnosis for the case vignettes, 9 (19.57%) had no change in knowledge, and knowledge got worse in 8 (17.39%). Amongst those who improved_,_ there was approximately a 100% increase in diagnostic accuracy (from mean accurate diagnoses (SD) of 2.03 (1.27) before the intervention, to 4 (0.96) after the intervention). Only pre-intervention knowledge (Odds Ratio (OR) = 0.217_,_95% CI = 0.085–0.552, *p* = 0.0013) and highest level of education (OR = 2.159_,_95% CI = 1.081–4.315, *p* = 0.029)_,_ were significant predictors of gains in accuracy in diagnosing disorders following the intervention. An increase in the highest Grade of education by one year was associated with more than doubling the odds of showing an improvement in correctly diagnosing the disorder in the vignettes, after adjusting for the other covariates. There was a marginally larger proportion of improvers at OTSM than Masincedane (64.3% versus 61.1%, Deviance score = 3.666_,_ *p* = 0.0555), with the site-effect probably due to the higher pre-intervention knowledge at OTSM. These differences in site were significant for pre-intervention knowledge only (Mann-Whitney Z = − 2.4325_,_ *p*-value = 0.015). Quantitative outcomes are presented Table 3.

**Table 3:** CHW Training Quantitative Outcomes

	Pre-training	Post-training			3 month post training		
Outcome	(mean, SD, N)	(mean, SD, N	Statistic	*p*-value	(mean, SD, N)	Statistic	*p*-value
Knowledge							
(MAKS)	41.48 (5.85), *N* = 58	45.57 (4.25) N = 56	*t* = −4.523 df = 55	< 0.001	45.67 (4.59) N = 54	*t* = −5.0 df = 53	*p* < 0.001
Confidence							
(MHNCCS)	45.25 (9.97), N = 58	61.75 (7.42) N = 54	*t* = −8.749 df = 54	< 0.001			
	Pre-training (mean, SD) *N* = 45	Post-training (mean, SD) *N* = 45					
Attitudes (CAMI)							
*Authoritarianism*	27.87 (2.97)	26.38 (4.1)	t = 2.720	0.99			
*Benevolence*	37.67 (4.46)	38.82 (3.79)	*t* = −1.818	0.04			
*Social Restrictiveness*	24.73 (4.28)	22.4 (5.3)	t = 2.96	0.002			
*Tolerance to rehabilitation in the community*	36.49 (5.11)	38.09 (4.22)	*t* = −2.18	0.02			

Analysis models are detailed in text

There was a significant increase in the average scores on the MAKS pre- to post-training for 56 participants (*t* = − 4.523, df = 55, *p* <  0.001). This difference remained when comparing the post-assessment and 3-month assessment scores (mean 45.67, SD 4.59, *t* = − 5.0, df = 53, *p* <  0.001, *N* = 54). Highest level of education was significantly associated with change in knowledge on the MAKS pre-and post the training (coeff = 1.106, *t* = 2.433, *p* = 0.019). An increase in the highest Grade of education by one year was associated with a relative increase by more than 1 point on changes in MAKS scores after adjusting for the other covariates.

#### Confidence

There was a significant increase in the average confidence scores pre-to post training for 54 participants (*t* = − 8.749, df = 54, p <  0.001) after 4 subjects were removed due to missing data. A qqplot indicated that distributional assumptions of normality were upheld. The only variable that was significantly associated with change in the confidence score pre-and post the training was the pre-training confidence score (coeff − 1.150, *t* = − 10.981, p <  0.001). A CHW with 1 extra point on the confidence scale at baseline demonstrated a reduction in change in confidence after the training by more than 1 point after adjusting for differences in age, education and years of service. A statistical trend was also observed for age (coeff = − 0.177, *t* = − 1.875, *p* = 0.067), with each additional year associated with a reduction in gains in confidence post training. A trend towards a significant effect on changes in confidence pre-post training was observed for having a medical condition (coeff = − 4.845, *t* = − 1.969, *p* = 0.055). Having a medical condition was associated with a decreased gain relative to someone without a condition in the effect of the training on confidence.

#### Attitudes

The CAMI data from 13 participants were removed, as a substantial amount of data was missing for the pre-training variables in particular. The total sample for the CAMI analyses was therefore 45. There was no evidence of a change in authoritarian cluster scores following the training (*t* = 2.720, *p*-value = 0.995), however there was some significant improvement in the rest of the attitude clusters.

Benevolence cluster scores increased significantly following training (*t* = − 1.818, df = 44, p-value = 0.0379). Only the corresponding pre-training benevolence score was significantly associated with change in scores pre-and post the training (coeff = − 0.597, *t* = − 4.919 *p* < 0.001), after adjusting for differences in age, education and years of service. An increase of one point at baseline was associated with a decrease by more than half a point in changes associated with the training. CHWs with their own medical conditions displayed a lower average change in scores than those without medical conditions (coeff = − 4.273, *t* = − 4.620, p < 0.001), after adjusting for pre-training scores and differences between groups in change scores. This was due to an increase in scores on this cluster for those without medical conditions only (mean = 2.19, SD = 4.25, *N* = 31), with minimal change observed in those with medical conditions (mean = − 1.14, SD = 3.39, *N* = 14). This was observed even though most of the people with medical conditions (13 of 14) were in the OTSM group, which actually demonstrated increased changes pre-post training. The change in score is substantially higher in the OTSM group (mean = 3.89, SD = 4.2, *N* = 18) than the Masincedane group (mean = − 0.15, SD = 3.16, *N* = 13) when people with medical conditions are removed.

There was a decrease in socially restrictive attitudes following training (*t* = 2.960, df = 44, *p* = 0.002). Only the corresponding pre-training score for this cluster was significantly associated with a pre- and post-training change in scores. An increase of one point at baseline was associated with a decrease by close to half a point in changes associated with the training (coeff = − 0.437, *t* = − 2.525, *p* = 0.016), after adjusting for differences in age, education and years of service. We noted a trend towards a reduction in scores post-training in CHW’s with more years of service (coeff = − 0.684, *t* = − 1.852, *p* = 0.071). Having a medical condition was also associated with a change in attitudes. CHW with medical problems changed their responses to a greater extent following the training (coeff = 3.2624, *t* = 2.083, *p* = 0.0436). A substantial reduction in score was only observed in CHWs without medical conditions (mean, SD = − 3.35, 5.17), compared to those with medical conditions (mean, SD = − 0.07, 4.98).

CHW’s were more tolerant to rehabilitation of mental health service users in the community following the training (*t* = − 2.176, df = 44, *p*-value = 0.018). The only variable that was significantly associated with change in this cluster of attitudes was the corresponding pre-training score, with an increase of 1 point at baseline being associated with a lesser increase in tolerance associated with the training (coeff = − 0.621, *t* = − 5.478, *p* < 0.001) after adjusting for differences in age, education and years of service. There was a sub-threshold effect for having a medical condition, with those with a condition demonstrating a reduced change in scores post training (coeff = − 2.105, *t* = − 1.760, *p* = 0.086).

### Training feedback

#### Daily evaluation questionnaires

Participants reported finding the training content easy to follow and understand. Themes emerging from the daily evaluation forms included 1) new exposure to aspects of culture; 2) lack of confidence in dealing with the mentally ill; 3) ongoing training needs, and 4) emphasizing positive features of the training; 5) expressing gratitude; and 6) ongoing training needs.

##### New exposure to aspects of culture

A few participants indicated that certain aspects of culture and cultural idioms that had been discussed were new to them and had not previously been fully understood.

##### Lack of confidence in dealing with the mentally ill

Two participants expressed uncertainty about the best way to interact with people with mental illness.

*“That people have that disease still looks normal and can do normal things still”.* (OTSM 30)

##### Emphasizing positive features of the training

Participants felt that the training had been presented in a clear and simple way by the facilitators. The information being presented was experienced as enjoyable, informative and interesting. CHWs could develop a better understanding regarding mental illness. Participants identified acquiring counselling skills as one of the main reasons they enjoyed the session. They reported benefitting most from (1) education on substance abuse, (2) additional psycho-education, (3) engagement and emotional involvement. They felt they had now been empowered to bring about a real difference as the training content was viewed as being important and applicable to the field of practice, and as such_,_ was viewed as likely to help the them make a meaningful contribution to their communities. Participants experienced it as helpful and informative to have the opportunity revise the content of previous sessions.

*“It is a very good lesson to us as we live in places where the is lot of people uses substance abuse but we couldn’t help because of lack of knowledge”* (OTSM 24).

Engagement and emotional involvement of the group was one of the reasons participants enjoyed the session. The sharing of their own narratives helped participants learn from each other.

“*By hearing different stories although some was very sad but at least I gain something and knowledge*”. (MS 13)

##### Expressing gratitude

The participants expressed their gratitude towards the facilitators for their efforts in presenting a session that was experienced as valuable and worthwhile.

##### Ongoing training needs

Participants indicated that more information was required about bipolar mood disorder, depression, and issues affecting the elderly. Others specified that more information regarding depression is needed while another felt that they would enjoy and benefit from longer sessions. Some participants reported they would like to get more education about substance use disorders, as a significant part of the CHWs field of work involved dealing with substance use disorders. Other participants reported experiencing the volume covered in the training overwhelming.

#### Training evaluation

##### Training content

The training programme was well received by the CHWs. They attributed their positive experience to several factors; including the accessibility and relevance of the training content, as well as the impact on their perceptions of mental health service users. Participants perceived the content of the training as informative and stimulating, as reported by participant OTSM 15: “*The eight week training was very informative. I have learnt quite a lot. Enjoyed every second*”. The content was recognized as highly applicable to their practice.

##### Delivery of training

The participants felt that the facilitator had created a comfortable environment and maintained clear communication. They experienced the use of relatable examples during the training programme as particularly helpful.

##### Suggestions for future training

A few participants felt that they would like to learn more about intellectual disability. Some participants commented that they wished the duration of the training was longer. One of the participants also suggested that information pamphlets should be handed out after the case studies.

## Discussion

Our main findings were 1) an overall improvement in knowledge as demonstrated by improved diagnostic accuracy and MAKS scores; 2) improvement in confidence and 3) an overall positive change in attitudes, amongst the trained CHWs in all but the authoritarianism subscale, 4) satisfaction with the content and processes of the training and expression of sentiments of gratitude and feeling empowered.

There is little work on the efficacy of community health worker training programmes [27]. The improvement in diagnostic accuracy at the end of this training programme is consistent with the findings in one similar study [16]. Previous work has suggested CHW performance is associated with age, sex, education level, years of service (experience), marital status, and financial status [28]. In our sample, higher pre-training knowledge reduced the likelihood of significant improvement in knowledge, while highest level of education was significantly associated with the likelihood of improvement in knowledge. This highlights the importance of taking into account basic training requirements and trainability for CHWs [29]. CHWs at one site showed marginally better improvement, possibly as a consequence of pre-training knowledge attained in the course of standard NPO training activities.

Confidence scores increased significantly following the training. There was an overall positive change in attitudes in all but the authoritarianism subscale. Higher scores on the authoritarian attitudes have previously been linked with prevailing societal stigma related to perceived danger from people with mental disorders [30]. This perceived danger is likely related to the perceived unpredictability and occasionally violent nature of psychiatric presentations [31]. The overall decrease in stigmatizing views and the improved confidence matched the findings of the abovementioned study [32]. Other studies of community and health worker attitudes towards the mentally ill have suggested that attitudes supporting social integration may be related to mental health training and experience, and linked to a grasp of the biopsychosocial causation of mental illness [33, 34]. The need for training that includes a focus on biopsychosocial causation was born out in a Nigerian study evaluating attitudes and beliefs about mental illness amongst church-based lay health workers [35]. Respondents in this study widely held stigmatizing culture-bound concepts as causative for mental illness. Our training began by engaging with such local beliefs before introducing the biopsychosocial discussion, and this appears to have gone some way towards improving attitudes amongst our participants. Having said this, it is worth noting that stigmatizing attitudes towards mental illness, such as authoritarianism, are difficult to change [36]. Much work is required to focus the research agenda in the field to adequately address this. CHWs having their own medical illness was associated with decreased gains in confidence and attitude. This finding suggests a need for more focused investigation of the impact that having a chronic medical illness may have on attitudes towards mental illness and mental health service users, as well as on confidence in executing support roles.

There were high level of satisfaction with the training. The qualitative assessment elicited themes of gratitude and appreciation for the processes and content of the training. CHWs felt that the training would assist them in improving the quality of their contribution to their communities. This altruistic sentiment of CHW’s desiring to do more for their communities has been noted before, where the Christian ethic of care alongside the concept of “Ubuntu”, which refers to the African concept of community, have navigated an uneasy tension with the CHWs own socioeconomic struggles and needs [37]. CHWs expressed areas in which further training would be desirable, and these included the areas of substance use disorders, bipolar mood disorder and geriatric psychiatry.

A few limitations of our investigation bear emphasizing. A first limitation is that we did not employ a randomized controlled design as this was a preliminary investigation, the findings of which might inform a future randomized controlled trial. The findings presented here must therefore be considered with caution. A second limitation is that we did not evaluate the impact of training on patient outcomes. While changes in knowledge, confidence and attitudes are necessary for improvements in care for mental health service users they are not necessarily a sufficient condition for improved community-based care for mental health service users. A third limitation is that our follow-up was only 3 months. Longer-term evaluation of outcomes are required to explore whether outcomes are sustained long-term.

## Conclusion

In conclusion, this community health worker training intervention found improvements in knowledge, confidence and attitudes amongst the trained CHWs. Participant feedback indicated that the training was acceptable, while high attendance and stakeholder buy-in pointed to feasibility and the potential for engaging CHWs in delivery task shifted mental health care. Further work is required to establish 1) how one might focus specifically on those CHWs who are lacking in confidence or 2) who have low starting knowledge, and those who are seen to have worsening knowledge after such a training. Further controlled trials are warranted to assess training and its impact on patient outcomes.

## Abbreviations

CAMI: Community Attitudes Towards The Mentally Ill Scale
CHW: Community Health Worker
MAKS: Mental Health Knowledge Schedule
MHNCCS: Mental Health Nursing Clinical Confidence Scale
NGO: Non-Governmental Organizations
OTSM: Opportunity To Serve Ministries
